# Health-related quality of life measurement in asthma and chronic obstructive pulmonary disease: review of the 2009-2014 literature

**DOI:** 10.1186/s40248-016-0040-9

**Published:** 2016-02-15

**Authors:** Fabio Arpinelli, Mauro Carone, Gioacchino Riccardo, Giorgio Bertolotti

**Affiliations:** GSK Medical and Scientific Department, Verona, Italy; Fondazione Salvatore Maugeri, IRCCS, Pneumology Division, Cassano Murge, Italy; Fondazione Salvatore Maugeri, IRCCS, Psychology Service, Tradate, Italy

**Keywords:** Asthma, COPD, Asthma quality of life questionnaire, Health-related quality of life, Saint George's respiratory questionnaire

## Abstract

**Background:**

Asthma and chronic obstructive pulmonary disease (COPD) are frequent in the general population. These diseases can worsen the quality of life of people suffering from them, limiting their daily activities and disrupting their sleep at night. Some questionnaires to measure the impact of the diseases on the daily life of patients are available. The measurements of subjective outcomes have become a part of clinical practice, and are used very frequently in clinical trials. Our aim was to describe how data on HRQoL in asthma and COPD are reported in papers published in the medical literature.

**Methods:**

We identified papers on the recent respiratory drugs (chemical, not biological), that reported the HRQoL measurement and that were published from 2009 to April 2014. We planned to describe data about HRQoL, and we had no intention of comparing the degree of efficacy of drugs.

**Results:**

The most used questionnaires are the Asthma Quality of Life Questionnaire (AQLQ) and the Saint George's Respiratory Questionnaire (SGRQ). These tools, administered at the baseline and at the end of the study (and interim evaluations in the longer studies) allowed for the identification of improvements as perceived by the patient after the treatment, even if in some cases these improvements were limited and not clinically relevant. Subjective measurements have always been placed among the secondary endpoints and the number of patients (estimated for the main endpoint) has often statistically overestimated the result. In addition, it is clear that subjective data is normally reported, but rarely commented on.

**Conclusions:**

There are some methodology aspects that should be discussed in more depth, for example the necessity to express variations in the subjective perception, not as *p*-value but as effect-size.

## Background

Asthma and chronic obstructive pulmonary disease (COPD) are frequent in the general population. The prevalence of asthma is estimated to be between 1 and 18 % of the population in the countries where it has been studied [1], whereas COPD affects about 6 % of the adult population, with a higher prevalence in older age groups [2]. These diseases are characterised by a narrowing of the bronchi associated with chronic inflammation. However, there are some differences between asthma and COPD in terms of onset age, causal factors, clinical aspects and impact on daily life. Functional measurements are used to measure severity and reversibility of the pulmonary obstruction. However, the relationship between pulmonary obstruction, dyspnea and impact of the disease on the daily life of the patient, is not necessarily linear [3]. The subjective data obtained from the patients can be useful for supplementing clinical and instrumental data and enabling the physician to identify any change (improvements or worsening) in the state of health, as perceived by the patient, thus allowing for an adjustment of the treatment. Questionnaires do exist and have been used for decades as they can gather data, in a standardised form, about the patients’ perception of the status of the disease and/or the received medical treatments. This information, reported directly by the patient without other people’s interpretation, about his/her well-being, behaviour and feelings as regards his/her state of health and the related treatments, is defined as Patient-Reported Outcome (PRO) [4]. The Health-related Quality of Life (HRQoL) is a well known PRO. We have had available for some time questionnaires that are used for measuring HRQoL. European and USA regulatory authorities have published official guidance documents that reflect their position on this topic. The USA Food and Drug Administration (FDA) prepared a formal “Guidance for Industry” which describes its position on the PROs and informs the pharmaceutical industry on how it intends to review and evaluate the PRO tools that are used to support label claims [5]. The European Medicine Agency (EMA) document points out the role that the HRQoL may play in the development process of a medicinal product, by simply providing some recommendations on their use [6]. Recently, a number of new medicinal products to be used alone or in fixed combinations have been registered for the treatment of asthma and/or COPD. In many clinical studies of these drugs, HRQoL measurements were included.

Our aim was to deepen the knowledge on how the measurement of HRQoL was carried out for supporting the registration of these drugs (medicinal products of chemical and not biological origin). We planned to review the papers in order to describe and comment on the used tools, the results of the measurements and the use of the HRQoL data that were obtained. We had no intention to comment on the degree of efficacy of the medicinal products being considered.

## Review

We took into consideration the respiratory drugs that were launched in the last years. We described randomised and controlled clinical trials of medicinal products developed for asthma and for COPD, published from 2009 to the spring of 2014. The year 2009 was chosen because in that year the National Health Service in England (NHS) made the decision to measure the impact, as perceived by the patient, of some therapeutic procedures [7]. Non-randomised studies, *post-hoc *analyses, meta-analyses and/or systematic reviews of respiratory medicinal products have not been taken into account. For each study, the design, sample size, use of tools for the measurement of PROs have been identified and the related data briefly described. No statistical analyses of any type were carried out; in fact this report consists exclusively in a description of the findings.

Thirty four articles, published in the considered period, were analysed (see Tables 1 and 2). Eleven studies were on asthma. The questionnaire used in these studies was the Asthma Quality of Life questionnaire (AQLQ). The AQLQ is a 32 items questionnaire in 4 domains (symptoms, activity limitation, emotional function and environmental stimuli). It is suitable for adult with asthma, that should respond on a 7 point scale. Psychometric tests show that a change in score of 0.5 is the smallest change that can be considered clinically important (this means that the patient appreciates the change in his/her health condition).

**Table 1:** Studies on asthma

Drugs	Baseline	Final score	Statistical significance^a^	Clinical significance	Reference
flut/form 500/20 μg	4.42 ± 0.89	5.34 ± 1.07	*P* = 0.036		Bodzenta-Lukaszyk et al. Resp Med 105 674-682 2011 [13]
flut + form 500 + 24 μg	4.57 ± 0.99	5.30 ± 1.00			
flut/form250/10 μg	Not reported	Mean improvement of 0.8 in both treatment groups	ns	55 %	Bodzenta-Lukaszyk et a.l Journal of Asthma 49(10), 1060-1070, 2012 [14]
bud/form400/12 μg				57.6 % ns	
flut/form combo	4.8 ± 1.1	5.5 ± 1.1	ns	Not available	Bodzenta-Lukaszyk et al. Current Medical Research and Opinion 29, 5 579-588, 2013 [15]
flut + form	4.9 ± 1.2	5.6 ± 1.0			
Tio 5 μg add on	4.8 in all groups	Change of 0.1 points for both active treatments compared with placebo	ns	Not available	Kerstjens et al. Journal of Allergy Clin Immunol 128, 2 308-314, 2011 [16]
Tio 10 μg add on					
Plac add on					
Trial 1				Not achieved	Kerstjens et al. New England Journal of Medicine 367:1198-1207, 2012 [17]
Tio 5 μg/die add on	4.6 ± 1.1	5.15	Ns		
plac add on	4.6 ± 1.1	5.1	Ns (*p* < 0.05 al week 24		
Trial 2					
Tio 5 μg/die add on	4.6 ± 1.0	5.1			
plac add on	4.7 ± 1.1	4.93			
Bud/formSMART	4.78	4.81	ns	Not available – measurement of Satisfaction with Asthma Treatment Questionnaire	Louis R et al. The International Journal of Clinical Practice 63, 10 1479-1488, 2009 [18]
Conventional best practice	4.78	4.82			
Tio		5.58	*P* = 0.01	Not available	Peters et al. New England Journal of Medicine 363 18 1715-1726, 2010 [19]
Double glucocorticoid	5.43 ± 1.05	5.48	*P* = 0.38		
salm		5.71	*P* < 0.001		
Flut fur/vil 200/25 μg	Not available	Baseline + 0.93 ± 0.065	ns	Not available	O’Byrne et al. Eur. Resp. Journal 43 773-782, 2014 [20]
Flut fur.200 μg		Baseline + 0.88 ± 0.071			
Flut prop.500 μg		Baseline + 0.90 ± 0.068			
Flut. fur/vil 100/25 μg	5.35	5.85	ns	46 %	Woodcock et al. Chest 144(4), 1222-1229, 2013 [21]
flut. pro/salm 250/50 μg	5.37	5.79		38 %	
salm 50 μg	5.175	Baseline + 0.280	ns	Not available	Bateman et al. J. Allergy Clin Immunol128, 315-322, 2011 [22]
tio 5 μg		Baseline + 0.131			
plac		Baseline + 0.039		Not available	
Adjustable bud/form	AQLQ	AQLQ	AQLQ	AQLQ	O’Connor et al. Journal of Asthma 47 217-223, 2010 [10]
Fixed bud/form	Not available	Not available	*P* < 0.04*	66.7 %	
Adjustable bud/form			Ns^b^	63.0 %	
				61.9 %	
				No clinical difference between groups	

^a^Between groups

^b^Fixed-dose regimen baseline vs end of treatment

*Adjustable dosing vs fixed dose regimen

*p* ≤ 0.002 vs fluticasone propionate/salmeterol

**Table 2:** Studies on COPD

Drugs	Baseline SGRQ	Final score	Statistical significance^a^	Clinical significance	Reference
Aclid 200 μg	45.9	-4.7 vs baseline	*P* = 0.013 vs plac.	49 %*	Kerwin E.M., et al. *COPD patients (ACCORD COPD I).* COPD 9 90-101, 2012 [23]
Aclid 400 μg	48.3	-4.5 vs baseline	*P* = 0.019 vs plac.	45 %	
plac	45.1	- 2 vs baseline		36 %	
Aclid 200 μg	46.3 ± 16.8	.-3.8 ± 1.1 vs plac	*P* < 0.001vs plac	56.0 %**	Jones P.W., et al. Eur Resp Journal 40 830-836, 2012 [11]
Aclid 400 μg	47.6 ± 17.7	-4.6 ± 1.1	*P* < 0.0001 vs	57.3 %**	
plac	45.1 ± 15.8		plac.	41.0 %	
Aclid 200 μg	48.5	-5.3 vs baseline	ns	41-6 % - 46.6 %	Gelb A.F., et al. Respiratory Medicine 107 1957-1965, 2013 [24]
Aclid 400 μg	49.8	-5.2 vs baseline		45.2 % - 49.1 %	
Glycopyr 50 μg	46.11	39.50	*P* = 0.004	56.8 %	D’Urzo A., et al. Respiratory Research 12 156, 2011 [25]
Plac	46.34	42.31		46.3 %	
				*P* = 0.006	
Glycopyr 50 μg	Not	-3.32 vs placebo	*P* < 0.001	54.3 %	Kerwin E., at al. European Respiratory Journal 40 1106-1114, 2012 [26]
Tio 18 μg	reported	-2.84 vs placebo	*P* = 0.014	59.4 %	
Plac				50.8 %	
Indac 300 μg	43	-4.7 vs placebo	*P* < 0.001 vs plac	Not reported	Dahl R. et al. Thorax 65 473-479, 2010 [27]
Indac 600 μg	44	-4.6 vs placebo			
Form	44	-4.0 vs placebo			
Plac	43				
Indac 150 μg	43 ± 18.6	-5.0 vs baseline	*P* < 0.001	52.8 %**	Kornmann O. et al. European Respiratory Journal 37 273-279, 2011 [28]
Salm 50 μg	44. ± 18.4	-4.1 vs baseline	*P* < 0.001	48.6 %***	
plac	44 ± 18.1			38.0 %	
Indac 150 μg	Not reported	-3.3 vs placebo****	*P* < 0.001 vs plac	-	Donohue J.F. et al. Am J Respir Crit Care Med 182 155-162, 2010 [29]
Indac 300 μg		-2.4 vs placebo	*P* < 0.01 vs plac		
Tio 18 μg		-1.0 vs placebo	Ns vs plac		
plac					
Indac 150 μg	42.3 ± 17.60	37.1 ± 0.56	*P* < 0.001	50.5 %	Buhl R. et al. Eur Respir J 38 797-803, 2011 [30]
Tio 18 μg	42.7 ± 18.04	39.2 ± 0.55		42.5 %	
				p ≤ 0.001	
Indac 150 μg	47.9	42.3	*P* = 0.73	49 %	Decramer M.L. et al. Lancet Respir Med 1 524-533, 2013 [31]
Tio 18 μg	48.7	42.2		49 &	
Tio 5 μg	Not reported	-4.7 vs baseline	*P* < 0.0001	49.5 %	Bateman E.D. et al. Respiratory Medicine 104, 1460-1472, 2010 [32]
P lac		-1.8 vs baseline		41.4 %	
				*P* < 0.0001	
Tio 18 μg emphysema	46.7 ± 3.0	39.4 ± 2.7	ns	Not reported	Fujimoto K. et al. International Journal of COPD 6, 219-227, 2011 [33]
Tio 18 μg non emphys	35.1 ± 6.4	26.9 ± 4.6			
Salm 50 μg emphysema	38.6 ± 3.5	33.0 ± 3.2			
Salm 50 μg nonemphys	37.5 ± 8.5	29.3 ± 7.4			
Tio 18 μg	46.1 ± 19.1	-4.5 vs baseline	*P* < 0.05	Not reported	Hoshino M. et al Respirology 16 95-101, 2011 [34]
Tio + Salm/flut 50/250 μg	42.7 ± 17.0	-10.2 vs baseline			
Umec 62.5 μg	Not reported	-3.14 vs baseline	*P* < 0.001 both doses of umeclidinium vs placebo	Not reported	Trivedi R. et al. Eur Respiratory J 43 72-81, 2014 [35]
Umec 125 μg		-6.12 vs baseline			
Plac		+4.75 vs baseline			
Beclom/form 100/6 μg	60.4 ± 19.5	-3.75 ± 13.91	ns	25.40 %	Calverley P.M.A. et al*. *Respiratory Medicine 104 1858-1868, 2010 [36]
Bud/form 200/6 μg	57.2 ± 18.6	-4.28 ± 11.92		21.90 %	
Form 12 μg	59.5 ± 20.2	-2.90 ± 13.28		25.30 %	
Tio + bud/form	Not reported	-3.8 vs baseline	*P* = 0.023	49.5 %	Welte T. et al. Am J Respir Crit Care Med 180 741-750, 2009 [37]
Tio + plac		-1.5 vs baseline		40.0 %	
				*P* = 0.016	
Bud/form 320/9 μg	55.9 (17.6)	-7.2 (1.18) vs bas.	ns		Sharafkhaneh A. et al. Respiratory Medicine 106, 2257-268 2012 [38]
Bud/form 160/9 μg	57.8 (16.7)	-5.5 (1.17) vs bas.			
Form 9 μg	58.6 (16.9)	-5.9 (1.17) vs bas.			
Indac/Glycopyr 110/50 μg	42.01	35.45	ns	55.5 %	Vogelmeier C.F. et al. Lancet Respir Med 1 51-60, 2013 [39]
Salm/flut 50/500 μg	42.72	36.68		49.1 %	
Indat/Glycopyr 110/50 μg	53 (18)	43.8	glycop/indacat	57 %	Wedzicha J.A. et al. Lancet Respir Med 1 199-209, 2013 [40]
Glycopyr 50 μg	52 (18)	45.8	*P* = 0.0067 e	52 %	
Tio 18 μg	52 (17)	46.0	*P* = 0.00037 vs competitors	51 %	
				Glycopir/indacat *p* = 0.055 e *p* = 0.051 vs competitors	
Indac 150 μg + Glycopyr 50 μg	Not reported	- 6.22 (11.47)	ns	56.5 %	Vincken W. et al. Int Journal of COPD 9 215-228, 2014 [41]
Indac 150 μg		- 4.13 (10.38) vs baseline		46.8 % ns	
Beclom/form 200/12 μg	47.0 (16.7)	-5.92	*P* = 0.08	45.0 %	Singh D. et al. BMC Pulmonary Medicine 14 43 2014 [42]
Flutic/salm 500/50 μg	45.2 (16.5)	-3.80		36.2 %	
				ns	
Umec/Vil 62.5/25 μg	Not reported	-8.07 (0.749)	p ≤ 0.001 vs	49 %	Donohue J.F. et al. Respiratory Medicine 107 1538-1546, 2013 [43]
Umec 62.5 μg		-7.25 (0.753)	placebo	44 %	
Vil25 μg		-7.75 (0.760)		48 %	
Plac		-2.56 (0.950) vs baseline		34 %	
Umec/Vil125/25 μg	Not reported	40.10 (0.665)	Combination	49 %	Celli B. et al Chest 145 (5) 981-991, 2014 [44]
Umec125 μg		43.38 (0.664)	p ≤ 0.001 vs umec e	40 %	
Vil 25 μg		42.82 (0.681)	*p* <0.01 vs	41 %	
Plac		43.69 (0.875)	vilanterol	37 %	

^a^Between groups

*p < 0.005 vs placebo

**p < 0.001 vs placebo

***p < 0.01

****p < 0.01 vs tiotropium

Twenty three studies were on COPD. The questionnaire they used was the Saint George's Respiratory Questionnaire (SGRQ). It is a 50 items questionnaire designed to measure impact on overall health, daily life and perceived well-being in patients with COPD. Higher scores indicate more limitations. A mean change score of −4 units is considered as clinically important.

Only 3 studies on asthma reported a change that was statistically significant (27 % of the studies), and only 2 (18 % of the studies) reported the percentage of patients who reached the clinical significance (at least 0.5 change from baseline). The clinical significance ranged between 55.0 and 66.7 % of patients. Among studies on COPD, 16 (69.5 %) reported a statistical significance and 17 (80 %) reported the percentage of patients with the clinical significance (at least −4 units from baseline). The clinical significance ranged between 21.9 and 59.4 % of patients.

Usually, the authors describe in a very short way HRQoL results, and poor details are reported.

## Conclusions

A description of the literature about respiratory, non-biological medicinal products, developed in the past few years, has allowed to highlight that the PRO measurement has been often included in the studies’ protocols. This is in line with the recognition by regulatory authorities, payers and pharmaceutical companies that measurement of the patient experience with the drug is increasingly important. In all the measurements observed, the subjective data was considered a secondary endpoint. This is consistent with the endpoint hierarchy established by EMA and the FDA which sees the physiological effect produced by the study drug as a priority, and relegates the PRO to a secondary endpoint.

The choice of tools to be used meets the specifications of the reference documents. AQLQ is used with the patients with asthma, whereas the SGRQ is used with the patients with COPD. Both tools have been well documented since their initial development, and are validated and available in many languages [8, 9]. In all the studies, the time frame for the administration of the questionnaires is specified. In the shorter studies, the measurements are reported at the baseline and at the end of the study. In longer studies, there can be interim measurements. It should be noted that the short duration of a study (in particular in the COPD case) is planned to allow for functional measurements, but it could not capture the impact that the drug has on the health, as perceived by the patient. For instance, Kerwin reports, in the conclusions of his study, that a duration above 12 weeks could have allowed the patients treated with aclidinium 400 μg to reach the Minimal Clinical Important Difference (MCID) in a significantly greater percentage compared with the patients receiving placebo [10]. A further aspect for consideration is the use of the placebo treatment arm. These patients may receive rescue treatment, and often the variation in the SGRQ score obtained with these patients is important, as is the percentage of responder patients (those who reach the MCID). Another aspect noted in some studies was that the improvement reported with the SGRQ was in contrast with the bronchodilator effect. For example, Jones et al point out this aspect in the placebo arm of their study where the spirometry values decline, but the patients report a better respiratory state of health [11].

The measurements are always accompanied by information on their statistical significance. With reference to this point, we noted that more COPD than asthma studies reported a statistical significance. In other words, it seems that COPD drugs are significantly more effective than asthma drugs. Furthermore, it is to consider that a number of COPD studies foresaw the analysis versus a placebo arm, and the number of patients could be excessive for the HRQoL measurement. With reference to the clinical significance, asthma patients have a higher percentage of patients who experienced a clinically important change than COPD patients (despite the lower number of measurements). We can speculate that asthma patients perceive better the changes given by drugs than COPD patients. On the other hand, asthmatics are generally younger than COPD patients, and suffer less from other diseases (comorbidities). However, the subjective nature of the measurements should lead to some considerations on the significance of the effects produced by the treatment. Usually, the variations in the scores obtained from the questionnaires on HRQoL are assessed on an effect-size basis, since a change produced by a treatment may be statistically significant (possibly because the number of patients calculated for the main endpoint is high), but it may not be perceived as clinically significant by the patients. In general, effect-size indexes have been developed for the exact purpose to avoid making incorrect inferences, due also to high sample sizes. In other words, since the above indicated indexes seek to square the results net of the effects of the sample (number of subjects), they should not be affected by this data. The effect-size indexes allow for an evaluation of the amplitude of an effect in each research design, but there always remains the difficulty of choosing the most appropriate index and interpreting the results. Perhaps the latter is the reason why measurement results are usually reported without comments. In some cases, PRO measurement data are included in the discussion, but in many other cases, they are only briefly mentioned.

Undoubtedly, the PRO adds knowledge to the drug. Within the respiratory area, there are many medicinal products available which have demonstrated their efficacy in intervening on the two main characteristics that define asthma and COPD, i.e. bronchoconstriction and chronic inflammation. However, only some of them provide information about the outcomes of treatment which may be important to patients. This data could represent a discriminatory factor in the choice of the medicinal products to be included into a formulary. In fact, a medicinal product that is perceived as useful by the patient could have more probability to be taken (good adherence to treatment) and this would maximise the financial investment of the third party payer. Among the cited studies, one has used the Onset of Effect Questionnaire (OEQ) to measure the preference of the patient with asthma of a certain bronchodilator versus another bronchodilator in the same pharmacological category, but slower in manifesting its effects [10]. The fact that this questionnaire was not used in other studies may mean that it is not important to measure this parameter, or that the questionnaire was used in that study just to reiterate that one of the drugs was faster in manifesting its effects.

Some studies, not included in this analysis because they were published before 2009, measured the preferences of the patients regarding the inhaler used. Dalby et al cite these experiences in one article that discusses the development of an inhaler [12]. This measurement can be useful also if the intent is to provide the patient with a treatment, that, on average, he/she will consider satisfactory and therefore, presumably, be more willing to adopt.

To conclude, the subjective measurements, initially reserved for patients with very serious diseases and a limited life expectancy, have become more commonly used even with patients suffering from chronic diseases. The fact that patients with asthma and COPD can have a longer life expectancy makes the quality of those years left to live very important. It is therefore desirable that the PRO measurements be increasingly used and become part of the daily routine of clinical practice. In this perspective, tools such as the SGRQs might be considered labour-intensive requiring too long for their completion and interpretation. To this end, some other easier and simpler tools could be proposed for development.

## Abbreviations

AQLQ: Asthma Quality of Life questionnaire
COPD: chronic obstructive pulmonary disease
EMA: European Medicine Agency
FDA: Food and Drug Administration
HRQoL: Health-related Quality of Life
MCID: minimal clinical important difference
OEQ: onset of effect questionnaire
PRO: patient-reported outcome
SGRQ: Saint George's Respiratory Questionnaire
